# Natural Astaxanthin Improves Testosterone Synthesis and Sperm Mitochondrial Function in Aging Roosters

**DOI:** 10.3390/antiox11091684

**Published:** 2022-08-29

**Authors:** Shan Gao, Bang-Xin Zhao, Cheng Long, Nuo Heng, Yong Guo, Xi-Hui Sheng, Xiang-Guo Wang, Kai Xing, Long-Fei Xiao, He-Min Ni, Shu-Geng Wu, Xiao-Long Qi

**Affiliations:** 1 Animal Science and Technology College, Beijing University of Agriculture, Beijing 102206, China; 2 Institute of Feed Research, Chinese Academy of Agricultural Sciences, Beijing 100081, China

**Keywords:** natural astaxanthin, testosterone synthesis, mitochondrial function, apoptosis factors, sperm

## Abstract

Spermatogenesis, sperm motility, and apoptosis are dependent on the regulation of glandular hormones and mitochondria. Natural astaxanthin (ASTA) has antioxidant, anti-inflammatory, and anti-apoptotic properties. The present study evaluates the effects of ASTA on testosterone synthesis and mitochondrial function in aging roosters. Jinghong No. 1 layer breeder roosters (*n* = 96, 53-week old) were fed a corn–soybean meal basal diet containing 0, 25, 50, or 100 mg/kg ASTA for 6 weeks. The levels of plasma reproductive hormones and the mRNA and protein levels of molecules related to testosterone synthesis were significantly improved (*p* < 0.05) in the testes of the ASTA group roosters. In addition, antioxidant activities and free radical scavenging abilities in roosters of the ASTA groups were higher than those of the control group (*p* < 0.05). Mitochondrial electron transport chain complexes activities and mitochondrial membrane potential in sperm increased linearly with dietary ASTA supplementation (*p* < 0.05). The levels of reactive oxygen species and apoptosis factors decreased in roosters of the ASTA groups (*p* < 0.05). Collectively, these results suggest that dietary ASTA may improve testosterone levels and reduce sperm apoptosis, which may be related to the upregulation of the testosterone synthesis pathway and the enhancement of mitochondrial function in aging roosters.

## 1. Introduction

Age is an important factor that affects the reproductive performance of layer breeder roosters. In aging roosters, a weakened antioxidant defense system is responsible for causing testicular damage and reducing semen quality. Testes, as genital organs, are mainly responsible for spermatogenesis and testosterone production. Spermatogenesis depends on the regulation of multiple gonadal hormones and is affected by aging [1]. Testosterone plays a crucial role in spermatogenesis, testicular development, and the maintenance of male characteristics [2,3]. Decreased testosterone levels are a direct consequence of impaired spermatogenesis in aging roosters; therefore, testosterone is considered an essential hormone for spermatogenesis [4]. Several key enzymes, including steroidogenic acute regulatory protein (*StAR*), cholesterol side-chain cleavage enzyme (*P450scc*), and 3β-hydroxysteroid dehydrogenase (*3β-HSD*), are required for testosterone synthesis [5]. It has been demonstrated that increased expression of *StAR*, *P450scc*, and *3β-HSD* results in improved reproductive performance in aging roosters [6]. Moreover, morphological changes occur in the testes during aging [7]. Therefore, strategies should be investigated to directly increase the testosterone levels in aging breeder roosters.

Mitochondria have a variety of physiological and reproductive functions [8]. Adenosine triphosphate (ATP) is the most common form of chemical energy in cells [9]. As a biomarker of sperm health and fertility [10], mitochondria play an important role in the linear motility, hyperactivation, and capacitation of sperms, acrosome reaction, and fertilization; thus, they are important for male fertility [11]. The decline in male reproductive performance may be linked to the deregulation of mitochondrial membrane potential (MMP). It has been demonstrated that the loss of MMP and decreased energy within the sperm may lead to a decline in sperm motility [12].

Apoptosis is a classic type of cell death that involves mitochondria. Free radicals are produced primarily by the mitochondria, and abnormal mitochondrial function or structure can lead to increased production of these radicals [13]. A recent study showed that reactive oxygen species (ROS) could induce apoptosis via a mitochondria-dependent pathway and that ROS promoted the permeabilization of the outer mitochondrial membrane [14]. MMP loss results in the release of pro-apoptotic factors, such as Ca^2+^, cytochrome c (*Cyt-c*), and caspases, thereby promoting apoptosis [15]. Therefore, a strategy must be developed to lessen the impact of aging on mitochondrial function in the sperm of breeding roosters.

Recently, several studies have investigated the use of natural antioxidant compounds to prevent reproductive decline in aging roosters [16,17]. Astaxanthin (ASTA) is a member of the xanthophylls family of carotenoids, which can be extracted from a variety of microorganisms, phytoplankton, and marine organisms [18]. Currently, as a natural antioxidant, ASTA has also been demonstrated to have higher antioxidant activity than α-carotene, lycopene, lutein, and β-carotene [19]. Additionally, ASTA is 550 times more efficient than vitamin E at scavenging singlet oxygens [20]. ASTA is a precursor of vitamin A, and its biological activities include antioxidant [21], anti-inflammatory [22], and anti-apoptotic properties, as well as the inhibition of lipid peroxidation [23]. Our previous study suggested that dietary ASTA could enhance sperm motility in aging roosters (an increase from 64.40% to 76.23%), which affects the expression of the MAPK/Nrf2 pathway, and reinforce the antioxidant defense system [24]. Our previous study revealed that sperm concentration and semen volume were also remarkably increased in animals with ASTA supplementation. Although testosterone level and mitochondrial function can have a beneficial effect on sperm quality, little research has been conducted on the effect of ASTA on semen quality.

Therefore, the objective of this study is to evaluate the effects of dietary supplementation with different concentrations of ASTA on testosterone synthesis, mitochondria-regulated energy supply, and the apoptotic pathway.

## 2. Materials and Methods

All experimental protocols were approved by the Animal Care and Use Committee of the Beijing University of Agriculture (Approval ID: BUA-zc-20200073).

### 2.1. Experimental Design

*Haematococcuspluvialis* containing 1.54% ASTA was purchased from Jingzhou Natural Astaxanthin Inc. (Jingzhou, China). A total of 96 53-week-old Jinghong No. 1 layer breeder roosters were randomly assigned to one of the four dietary treatments, i.e., 0, 25, 50, or 100 mg/kg ASTA. Six replicates per treatment, with four birds per cage (70 × 60 × 75 cm), were used. All birds were fed a basal diet for 1 week and then assigned to a corn/soybean-meal-based diet containing various doses of ASTA for 6 weeks. The composition and nutrient levels of the corn–soybean meal-based diet are shown in Table 1.

### 2.2. Sample Collection

Each bird was trained to have semen collected every two weeks. One bird from each replicate was randomly selected for semen collection after the 6-week feeding trial. Semen samples were temporarily stored in a pre-heated (37 °C) water bath until analysis. One bird was randomly selected from each replicate and euthanized via cervical dislocation. Blood samples were collected from the left jugular vein, and plasma was separated by centrifugation at 3000× *g* for 10 min and stored at −20 °C until analysis of reproductive hormones. Immediately after euthanasia, the left testis was fixed in 4% paraformaldehyde and embedded in paraffin, and the remaining testis was removed and quickly frozen at −80 °C for later analysis.

### 2.3. Reproductive Hormones Analysis

The concentrations of luteinizing hormone (LH, H206), follicle-stimulating hormone (FSH, H101), and testosterone (H090) were measured using ELISA kits (Nanjing Jiancheng Bioengineering Institute, Nanjing, China) according to the manufacturer’s instructions. All assays were performed in 96-well plates, and the absorbance was measured at 450 nm (Thermo Fisher Scientific, Waltham, MA, USA). The hormone concentrations were determined by referring to a standard curve.

### 2.4. Antioxidant Enzymes and Free Oxygen Radical Analysis

The activities of antioxidant enzymes, including glutathione peroxidase (GSH-Px, A005-1), superoxide dismutase (SOD, A001-1), catalase (CAT, A007-1), and the abilities of scavenging hydroxyl radical (A018-1), superoxide anion (A052-1), total antioxidant capacity (T-AOC, A015-1), and malondialdehyde (MDA, A003-1) were analyzed in sperm using a commercial kit (Nanjing Jiancheng Bioengineering Institute, Nanjing, China). The methods and principles used to determine the levels of these antioxidant indicators using these kits have been described previously [25].

### 2.5. Sperm Mitochondrial Preparation

A mitochondria extraction kit (Nanjing Jian Cheng Bioengineering Institute, Nanjing, China, G006) was used to extract sperm mitochondria in accordance with the manufacturer’s instructions. Semen (0.3 mL) was centrifuged for 10 min at 600× *g* and 4 °C. The semen plasma was eliminated, the sperms were washed three times with phosphate-buffered saline (PBS), and the supernatants were discarded. The spermatozoa were resuspended in buffer, and the homogenate was centrifuged for 5 min at 800× *g* at 4 °C. The supernatant containing mitochondria was placed in a pre-cooled centrifuge tube and centrifuged at 15,000× *g* for 10 min at 4 °C; the precipitate was the sperm mitochondria. The mitochondrial precipitate was lysed for 15 min using a lysis buffer in an ice-water bath with shaking. The mixture was then centrifuged for 15 min at 12,000× *g* and 4 °C. Mitochondrial protein concentration was determined using a bicinchoninic acid (BCA) protein assay kit (Nanjing Jiancheng Bioengineering Institute, Nanjing, China, A045-4).

### 2.6. Oxidative Phosphorylation (OXPHOS) Complexes Analysis

The activities of sperm mitochondrial respiratory chain complex I (NADH ubiquinone oxidoreductase, A089-1), complex II (succinate dehydrogenase, A089-2), complex III (ubiquinol cytochrome C reductase, A089-3), complex IV (cytochrome C oxidase, A089-4), and complex V (ATP synthase, A089-5) were evaluated in accordance with the manufacturer’s instructions (Nanjing Jiancheng Bioengineering Institute, Nanjing, China). The activity of complex I was evaluated following the oxidation of NADH at 340 nm. The activity of complex II was quantitatively determined using oxidized dichlorophenol indophenol to reduce dichlorophenol indophenol at 600 nm. The activity of complex III was evaluated by measuring the degree of reduction of *Cyt-c* by the enzyme at 550 nm. The activity of complex IV was evaluated by measuring the oxidation of *Cyt-c* for 60 s at 550 nm. Mitochondrial complex V activity was measured using the ultimate reaction of OXPHOS. In reactions catalyzed by pyruvate kinase (PK) and lactate dehydrogenase (LDH), the conversion of nicotinamide adenine dinucleotide (NAD), resulting in a change in the absorbance peak (340 nm), was quantified to analyze complex V activity.

### 2.7. Sperm MMP, ROS Production, and Apoptosis Analysis

The semen samples were diluted with PBS (approximately 1 × 10^6^ mL). Flow cytometry (Becton Dickinson, CA, USA) was used to detect MMP, ROS production, and apoptosis in at least 10,000 sperm cells. MMP was evaluated using a MitoProbe™ JC-1 Assay Kit (Thermo Fisher Scientific, Shanghai, China, M34652), which included a cationic dye, JC-1 (5′,6,6′-tetrachloro-1,1′,3,3′-tetraethylbenzimidazolylcarbocyanine iodide). Sperm were incubated with JC-1 in the dark for 30 min at 37 °C. They were then washed with PBS and analyzed. Potential-dependent accumulation of JC-1 in the mitochondria can be observed by a shift in fluorescence emission from green (529 nm) to red (590 nm). A decrease in the red/green fluorescence intensity ratio indicates mitochondrial depolarization. Thus, the MMP (high, H-MMP, and low, L-MMP) (ratio) of the samples was determined.

An ROS assay kit (Beyotime Institute of Biotechnology, Shanghai, China, S0033) was used to measure ROS production, which included a fluorescent molecular probe and 2,7-dichlorodi-hydrofluorescein diacetate (DCFH-DA). Sperm were incubated with 10 μM DCFH-DA in the dark for 20 min at 37 °C. The samples were washed thrice with PBS prior to flow cytometry. All flow cytometry analyses were performed using a 488 nm excitation laser and a 525 nm emission laser. After obtaining the green fluorescence signal, the positive rate of ROS and the mean fluorescence intensity were evaluated.

Apoptosis was analyzed using an FITC-Annexin V Apoptosis Detection Kit I (Nanjing Jiancheng Bioengineering Institute, Nanjing, China, 556547). Sperm samples were collected, washed with PBS, and resuspended in 1× binding buffer. They were then incubated with 5 μL PI, 5 μL FITC-Annexin V (dark, 37 °C, 15 min), and 1× binding buffer. Sperm apoptosis was analyzed by flow cytometry.

### 2.8. Caspase Activity Analysis

The levels of caspase-3 and caspase-9 were measured using kits (Beyotime Institute of Biotechnology, Shanghai, China, C1115 and C1157). Testis tissue samples (0.1 g) were homogenized in pre-cooled (4 °C) saline (0.9%, 1:9), and the levels of caspase-3 and caspase-9 were calculated based on the cleavage of the specific indicator fluorogenic substrates (AC-DEVD-pNA for caspase-3 and AC-LEHD-pNA for caspase-9). Substrate cleavage was determined at an excitation wavelength of 405 nm using a fluorescence spectrophotometer 405 nm excitation wavelength.

### 2.9. Testes Histology Analysis

After the left testis sample was fixed in 4% paraformaldehyde for 48 h, at least six sections from the testis samples were embedded in paraffin blocks and sectioned into 5 µm thick sections for histological examination. Three seminiferous tubules with regular contours were randomly analyzed in each section. The thickness of the seminiferous epithelium and the diameter of the seminiferous tubule were measured using Image-Pro-Plus (Media Cybernetics Inc., Rockville, MD, USA) at 100× magnification.

### 2.10. Quantitative Real-Time PCR (qRT-PCR) Analysis

Total RNA was extracted from a 0.1 g testis sample using TRIzol reagent (Thermo Fisher Scientific, Shanghai, China), as per the manufacturer’s instructions, and then reverse-transcribed into single-stranded cDNA using the Thermo First cDNA Synthesis Kit (Promega, Beijing, China). The expressions of *3β-HSD*, *P450scc*, *StAR*, *Caspase-3*, *Caspase-9*, *Bax*, *Bcl-2*, and *Cyt-c* were determined using qRT-PCR with specific primers (Table 2). After initial denaturation at 95 °C for 10 min, 40 cycles of amplification were carried out (95 °C for 10 s and 58.2 °C for 30 s), followed by the generation of melt curves that could be used to verify the specificity of amplification. Relative gene expression was calculated using the 2^−ΔΔCt^ method [26], with *ACTB* serving as the reference gene.

### 2.11. Western Blotting

Testes samples were lysed in radio immunoprecipitation assay (RIPA) buffer (SinoGene, Beijing, China) supplemented with proteinase inhibitors for 20 min, in accordance with the manufacturer’s instructions. The Bradford method was used to determine protein concentrations (SinoGene, Beijing, China). Protein markers (Thermo Fisher Scientific, Shanghai, China) and samples containing 30 μg of protein were separated using SDS-PAGE gels and transferred onto polyvinylidene difluoride (PVDF) membranes using a Bio-Rad mini transfer system (Bio-Rad Laboratories, Hercules, CA, USA) at 120 V for 2 h. Membranes were then incubated overnight with primary antibodies against β-actin (ABclonal Technology Co.,Ltd., Wuhan, China, AC028, 1:3000), anti-3β-HSD (Bioss lnc., Boston, MA, USA, bs-3906R, 1:1000), anti-P450scc (Bioss, bs-10099R, 1:1000), and anti-StAR (Abcam plc., Cambridge, Cambridgeshire CB2 0AX, UK, ab133657, 1:2000) at 4 °C. After being rinsed four times with Tris-buffered saline containing 0.1% Tween^®^ 20 Detergent (TBST) (10 min per rinse), the membranes were incubated with the corresponding HRP-conjugated secondary antibodies for 1 h. A chemiluminescent (ECL) kit (Engreen, Biosystem, Ltd., Beijing, China) was used to detect the immunological signals in the dark. Protein bands were quantified through densitometric analysis using Image J (National Institutes of Health, Baltimore, MD, USA).

### 2.12. Statistical Analysis

All statistical analyses were performed using SPSS 22.0 (IBM Corp., Armonk, NY, USA). Data are presented as mean ± standard deviation of the mean (SD). Additionally, polynomial regression analysis was used to test the linear and quadratic nature of the response to the additive ASTA dosage, and Tukey’s multiple comparison tests were used to analyze the differences among various treatments. *p* < 0.05 was considered significant.

## 3. Results

### 3.1. Reproductive Hormones Level and Testes Histology

**Figure 1:**
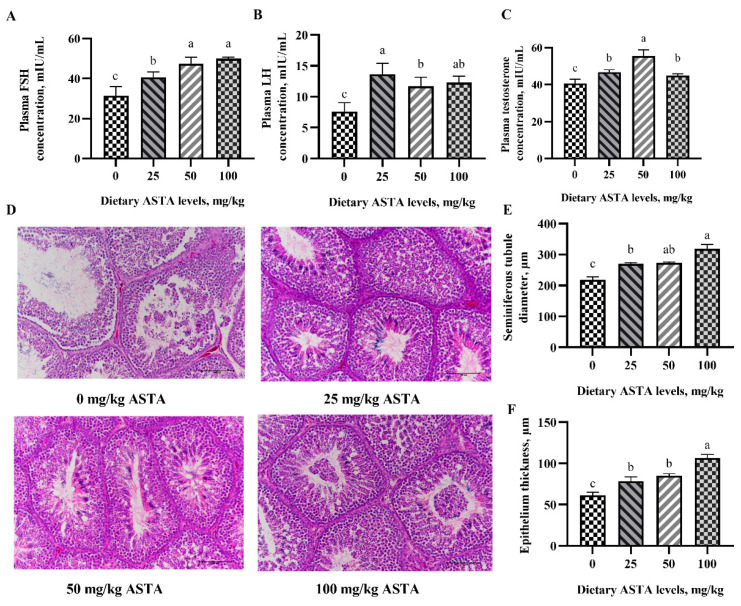
Effect of dietary ASTA supplementation on the plasma reproductive hormones and testicular histological parameters of aging roosters. (**A**) FSH level, (**B**) LH level, (**C**) testosterone level, (**D**) testicular morphology, (**E**) seminiferous tubule diameter, and (**F**) epithelium thickness. The values are expressed as means ± standard deviation, *n* = 6 in each group. ^a–c^ Means within a row with no common superscripts are significantly different (*p* < 0.05).

Testosterone is a key hormone related to spermatogenesis and metabolism, whose secretion is stimulated by FSH and LH. Generally, LH activates the protein kinase A (PKA) pathway and promotes the expression of key steroid genes by stimulating G proteins and adenylyl cyclase. Figure 1A–C show the effect of dietary ASTA supplementation on the levels of reproductive hormones in the plasma. When the concentration of ASTA reached 50 mg/kg, the FSH and testosterone levels increased remarkably compared to the control group (*p* < 0.05). In the group with 25 mg/kg ASTA supplementation, LH levels were significantly higher compared to the control group and reached a maxima (*p* < 0.05). The results also showed that the diameter of the seminiferous tubules and thickness of the epithelium were markedly increased in the ASTA groups (*p* < 0.05) (Figure 1D–F).

### 3.2. Expression of Genes Involved in the Testosterone Synthesis Pathway at the mRNA and Protein Levels

**Figure 2:**
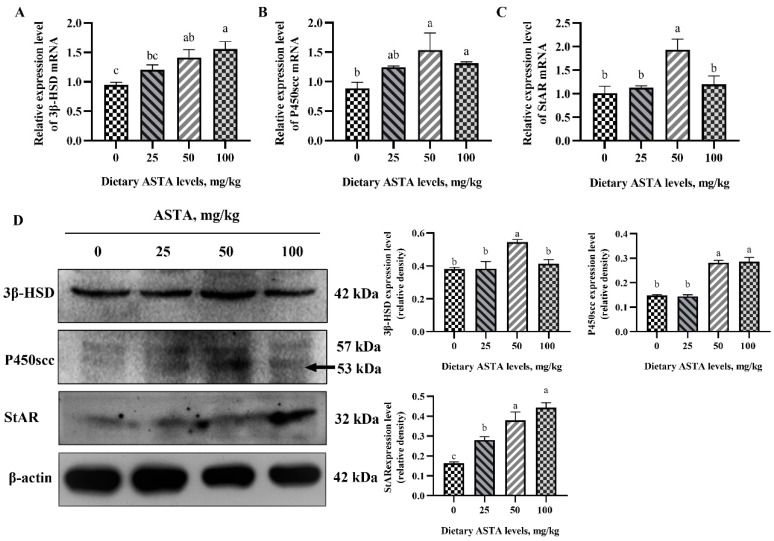
Effect of dietary ASTA supplementation on the expression of testosterone synthesis pathway intermediaries at the mRNA and protein level in the testes of aging roosters. (**A**–**C**) The expression of testosterone synthesis pathway intermediaries at the mRNA level; the values are expressed as means ± standard deviation, *n* = 6 in each group. (**D**) The expression of testosterone synthesis pathway intermediaries at the protein level; the results are expressed as mean ± standard deviation (*n* = 3). ^a–c^ Means within a row with no common superscripts are significantly different (*p* < 0.05).

The mRNA and protein levels of the testosterone synthesis pathway intermediaries in the testes of aged roosters are shown in Figure 2. When the ASTA concentration reached 100 mg/kg, the expression of 3β-HSD at the mRNA and protein level was significantly higher than that in the control group (*p* < 0.05). Further, in roosters of the 50 mg/kg ASTA group, the expression of P450scc and StAR at the mRNA and protein level was increased markedly in relation to the control group (*p* < 0.05). However, there was no significant difference in expression of P450scc at the mRNA and protein level in roosters supplemented with 50 and 100 mg/kg ASTA.

### 3.3. Antioxidant Ability of Sperm

**Figure 3:**
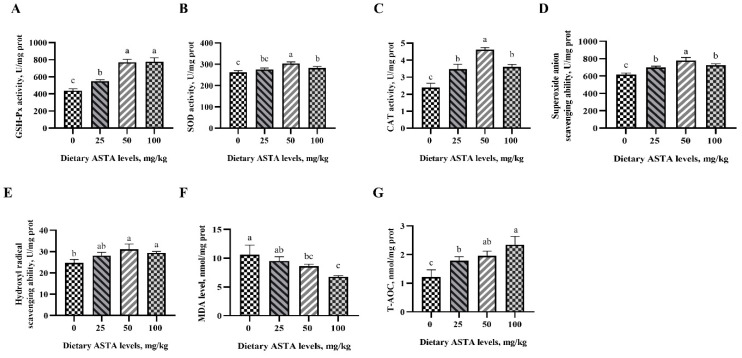
Effect of dietary ASTA supplementation on antioxidant enzyme activity and free radicals in the sperm of aging layer breeder roosters. (**A**) GSH-Px activity, (**B**) SOD activity, (**C**) CAT activity, (**D**,**E**) scavenging free radical ability, (**F**) MDA level, and (**G**) T-AOC ability. The data represent mean ± standard deviation; *n* = 6 in each group. ^a–c^ Means within a row with no common superscripts are significantly different (*p* < 0.05).

Antioxidant capacity can maintain sperm motility and viability. We investigated the effects of dietary ASTA supplementation on the antioxidant enzyme activity and free radical scavenging ability in rooster sperms (Figure 3). In roosters of the ASTA groups, sperm GSH-Px activity was markedly higher than in roosters of the control group (*p* < 0.05). SOD and CAT activities were higher in roosters of the 50 mg/kg ASTA group (*p* < 0.05). T-AOC levels were the highest in the sperm of roosters supplemented with 100 mg/kg ASTA (*p* < 0.05), while the MDA levels decreased linearly (*p* < 0.05). Notably, 50 mg/kg ASTA was better at enhancing superoxide anion scavenging ability than the other ASTA doses (*p* < 0.05). Additionally, the hydroxyl radical scavenging ability of roosters in the 50 and 100 mg/kg ASTA groups was significantly higher than that in roosters of the control group (*p* < 0.05).

### 3.4. OXPHOS Complex Activity

**Figure 4:**
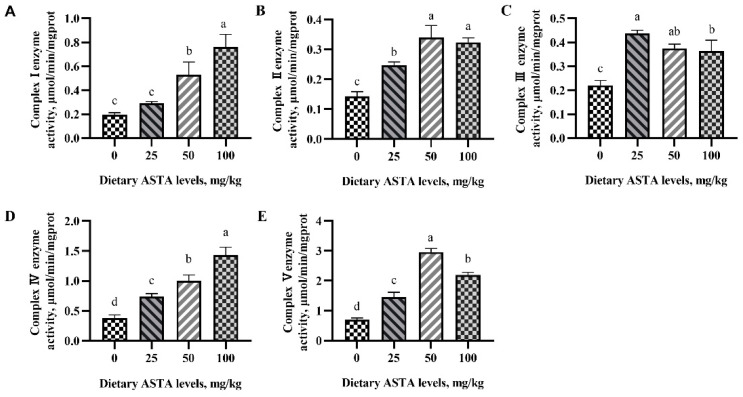
Effect of dietary ASTA supplementation on the activities of OXPHOS complexes. (**A**) NADH ubiquinone oxidoreductase (complex I), (**B**) succinate dehydrogenase (complex II), (**C**) ubiquinol cytochrome C reductase (complex III), (**D**) cytochrome C oxidase (complex IV), and (**E**) ATP synthase (complex V). The data represent mean ± standard deviation; *n* = 3 in each group. ^a–d^ Means within a row with no common superscripts are significantly different (*p* < 0.05).

OXPHOS complex activity is important for mitochondrial ATP production. In the present study, we evaluated the effects of dietary ASTA supplementation on the activity of OXPHOS complexes in sperms (Figure 4). Dietary addition of 25, 50, and 100 mg/kg ASTA resulted in markedly enhanced activities of complexes I–V (*p* < 0.05). Briefly, complex I activity increased in roosters of the 50 and 100 mg/kg ASTA groups relative to that in roosters of the control group (*p* < 0.05). The activities of complexes II and V in roosters of the 50 mg/kg AST group were higher than those in roosters of the other groups (*p* < 0.05). When the concentration of dietary ASTA reached 25 mg/kg, the activity of complex III was significantly higher than that in the control group (*p* < 0.05) and reached the maxima. Meanwhile, an increase in dietary ASTA supplementation from 25 to 100 mg/kg markedly enhanced (*p* < 0.05) the activity of complex IV compared to the control group.

### 3.5. MMP, ROS, and Apoptosis Levels

**Figure 5:**
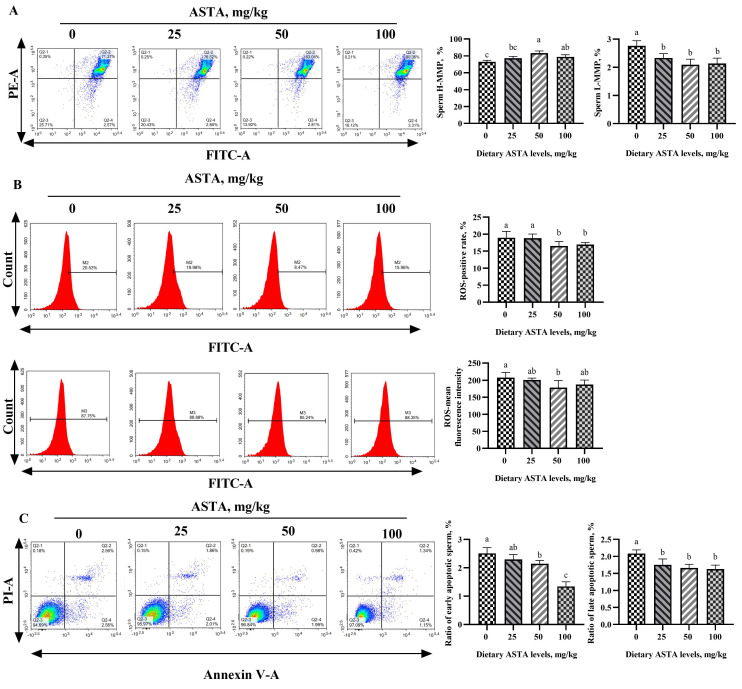
Effect of dietary ASTA supplementation on the level of MMP, ROS, and apoptosis of sperm. (**A**) MMP levels of sperm are measured by flow cytometry assay, (**B**) ROS level, and (**C**) sperm apoptosis level. The data represent mean ± standard deviation; *n* = 6 in each group. ^a–c^ Means within a row with no common superscripts are significantly different (*p* < 0.05).

The effects of dietary ASTA supplementation on MMP, ROS, and sperm apoptosis levels are shown in Figure 5. The percentage of sperm with H-MMP in roosters of the 50 mg/kg ASTA group remarkably improved compared to that in roosters of the control group, and the percentage of sperm with L-MMP decreased with ASTA supplementation (*p* < 0.05). In addition, the positivity and mean fluorescence intensity corresponding to ROS and sperm apoptotic levels markedly decreased in roosters of the ASTA groups (*p* < 0.05). In particular, when compared to the control group, the ratio of early apoptotic sperm gradually decreased with increasing dietary ASTA supplementation from 50 to 100 mg/kg (*p* < 0.05).

### 3.6. Caspase-3 and Caspase-9 Activation and the mRNA Expression of Apoptosis Factors

**Figure 6:**
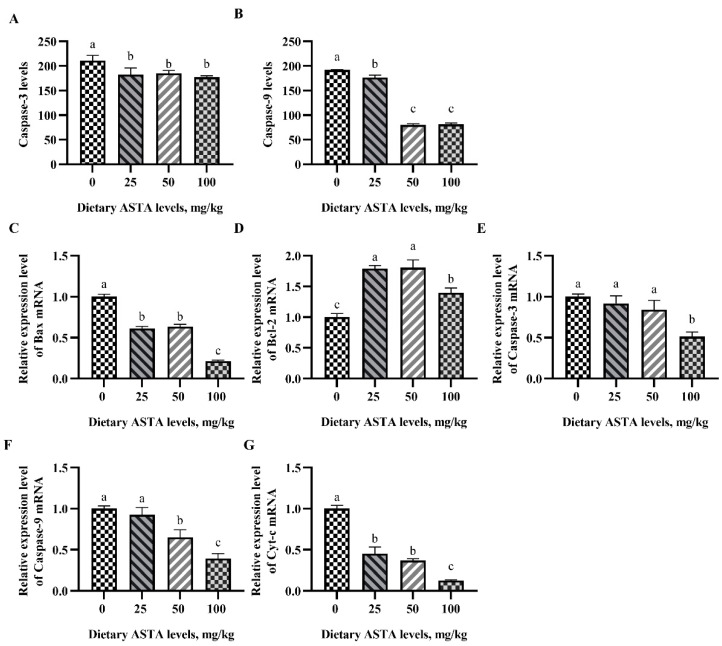
Effect of dietary ASTA supplementation on apoptotic factors in the testes of aging roosters. (**A**) Caspase-3 level and (**B**) caspase-9 level. The mRNA expression of (**C**) *Bax*, (**D**) *Bcl-2*, (**E**) *Caspase-3*, (**F**) *Caspase-9*, and (**G**) *Cyt-c* relative to that of β-actin (*ACTB*). The data represent the mean ± standard deviation; *n* = 6 in each group. ^a–c^ Means within a row with no common superscripts are significantly different (*p* < 0.05).

The effects of dietary ASTA on caspase-3 and caspase -9 activity are shown in Figure 6A,B. Dietary supplementation with ASTA significantly decreased caspase-3 levels in testes (*p* < 0.05). In addition, caspase-9 levels decreased gradually with increasing dietary ASTA supplementation from 25 to 100 mg/kg (*p* < 0.05), while no significant differences in caspase-9 levels were observed in roosters of the 50 and 100 mg/kg ASTA groups. To clarify whether ASTA affects the expression of apoptotic factors, the transcript-level expression of *caspase-3*, *caspase-9*, *Cyt-c*, *Bcl-2*, and *Bax* were measured in the testes (see Figure 6C–G). The transcript-level expression of *caspase-3* and *Caspase-9* remarkably decreased with dietary supplementation of 100 mg/kg ASTA (*p* < 0.05) and reached a minima. The levels of *Bcl-2* mRNA in roosters of the 50 mg/kg ASTA group were higher than those in the other groups (*p* < 0.05), whereas the levels of *Cyt-c* and *Bax* mRNA were significantly decreased in roosters of the 25 to 100 mg/kg ASTA groups (*p* < 0.05).

## 4. Discussion

Testosterone synthesis requires the conversion of cholesterol in the testes through a complex series of chemical reactions. StAR regulates the acute transport of cholesterol from the outer to the inner membrane of mitochondria in the testes. As P450scc catalyzes the conversion of cholesterol into 22R-hydroxycholesterol in the inner mitochondrial membrane, the double bond of 22R-hydroxycholesterol is cleaved to produce pregnenolone [27]. Finally, 3β-HSD catalyzes pregnenolone to testosterone in the endoplasmic reticulum [28]. Studies have shown that dietary antioxidant supplementation with molecules such as L-carnitine, soybean isoflavones, and linseed oil aids the synthesis and secretion of reproductive hormones [29,30,31]. These results indicate that dietary supplementation with ASTA, a natural antioxidant, may aid the synthesis and secretion of reproductive hormones. Testicular oxidative stress may be one of the major factors that reduce testosterone production. The major factor in the generation of oxidative stress is an imbalance between ROS production and the scavenging capacity of the cellular antioxidant defense system [32]. As mitochondria are critical sites for steroid hormone biosynthesis, ROS inhibit P450scc and StAR activity and function, leading to decreased testosterone production [33,34]. In addition, MMP loss induced by ROS also causes the downregulation of testosterone synthesis [35]. It has been reported that following hydrogen-peroxide–induced oxidative stress in Leydig cells, ASTA could reduce ROS production, prevent downregulated expression of the mature form of the StAR protein, and restore steroidogenesis [36]. Thus, in the current study, we investigated our hypothesis that ASTA, as an antioxidant, can improve testosterone production by decreasing mitochondrial ROS levels in aging roosters.

It has been shown that antioxidants, including enzymatic or non-enzymatic compounds, can improve the antioxidant defense system and play a key role in the prevention and treatment of diseases [37]. In a previous study, dietary supplementation with ASTA was found to improve SOD and GSH-Px activities and reduce lipid peroxidation in laying hens [38]. Therefore, we hypothesized that ASTA could enhance semen quality, possibly by improving the redox balance and membrane protection of sperm and decreasing MDA levels. The effective antioxidant activity of ASTA can be attributed to the conjugated polyene and terminal ring of ASTA, which capture free radicals on the surface and within the membrane, respectively [39]. These results suggest that dietary ASTA exhibits an antioxidative function in sperm by scavenging and neutralizing free radicals before they can damage the sperm membrane. Although dietary supplementation with ASTA is beneficial for sperm antioxidant capacity, whether ASTA can improve sperm mitochondrial function by improving the sperm antioxidant capacity in aging roosters remains unknown.

The outer mitochondrial membrane enwraps the inner membrane, which allows ions and most metabolites less than 10 kDa in size to enter the mitochondria [11,40]. The inner mitochondrial membrane has a larger surface area than the outer membrane, owing to its numerous cristae or invaginations, which increase the surface area that can produce energy via OXPHOS [41]. OXPHOS respiratory chain complexes contain a number of small soluble electron carrier proteins and cytochrome c, which are located on the mitochondrial cristae membrane [42]. Mitochondrial OXPHOS is a complex process that requires the coordinated operation of the respiratory chain and ATP synthases. The mitochondrial respiratory chain is composed of four complexes (complex I, complex II, complex III, and complex IV), which can actively transfer protons into the inner mitochondrial membrane, resulting in transmembrane differences in the proton gradient [43]. Finally, ATP synthase uses the free energy generated by the physiological dissipation of the proton motive force on the inner membrane of mitochondria to synthesize ATP and provides energy for cells [44]. To enable electron transport, a variety of protein carriers are present in the lipid bilayer of the inner mitochondrial membrane, including the ATP carrier [45] and the phosphate carrier [46], which are necessary for ATP synthesis inside the mitochondria. Progressive sperm motility largely depends on energy production from the mitochondrial compartment, which supports the basic role of mitochondrial phosphorylation in sperm movement [47]. Another study showed that sperm motility is strongly dependent on the function of the OXPHOS pathway, indicating that sperm motility is directly related to the activity of complexes I–IV [48]. Our results suggest that dietary ASTA successfully improves the activity of these complexes in the sperm. Therefore, we suspected that ASTA might improve the activity of OXPHOS complexes in two ways. First, ASTA has a special molecular structure that may physically affect mitochondrial membrane dynamics. For example, ASTA may affect the function of the mitochondrial membrane and membrane proteins (such as components of the electron transport chain (ETC)) through a special signal transduction mechanism, i.e., ASTA can regulate the association between adapter proteins and receptors in the plasma membrane. In the next step, ASTA cannot reduce the ROS content during ETC; it only neutralizes ROS and promotes ATP production during this process. However, strategies to improve OXPHOS activity need further exploration.

Superoxide anions are mainly produced in respiratory complexes I and III of the mitochondrial ETC; thus, in the sperm, ROS mainly originate from the mitochondria [49]. We speculated that ASTA has both lipophilic and hydrophilic properties, which can directly act on mitochondria through cell uptake and improve MMP. These results are similar to those reported in a previous study [50]. ROS, a byproduct of oxidative phosphorylation, is necessary for proper sperm function. Low ROS levels can promote sperm tyrosine phosphorylation, sperm hyperactivation, and sperm–egg interaction, but excessive ROS levels lead to oxidative stress and affect sperm function [51]. A study has shown that mitochondrial ROS levels correlate positively with lipid peroxidation, whereas ROS levels and MMP are negatively correlated [52]. A recent study showed that ASTA could effectively improve mitochondrial function by maintaining the MMP and reducing the ROS content [53]. Therefore, we suggest that dietary supplementation with ASTA reduces ROS levels and inhibits sperm apoptosis. These findings also confirmed our hypothesis that dietary ASTA increases the activities of key proteins involved in the testosterone synthesis pathway by increasing the MMP and reducing ROS production.

Apoptosis is a complex cascade of event responses; thus, evaluation of the effects of dietary ASTA on apoptosis factors in aging roosters is necessary. Numerous pathways regulate apoptosis, including intrinsic (mitochondrial), extrinsic, granzyme-mediated [54], and endoplasmic reticulum stress-mediated pathways [55]. The intrinsic pathway is regulated by the pro-apoptotic BAX-BAK and anti-apoptotic BCL-2 and BCL-XL proteins [56]. Mitochondria have been linked to apoptosis for years. They are the source of caspase cascades that result in protein degradation and apoptosis [57]. Caspase 9 is a key effector caspase in the intrinsic apoptosis pathway, and Cyt-c is a protein released into the mitochondrial inner membrane. However, whether dietary ASTA may play a role in the intrinsic mitochondria-dependent pathway in aging roosters remains unclear. Therefore, we evaluated the effects of dietary ASTA supplementation on caspase-3 and caspase-9 activity and the expression of apoptosis factors in the testes of aging roosters. The current results are similar to those obtained in previous studies, which also showed that ASTA inhibits the activation of caspase-3 and caspase-9 and the overproduction of ROS in cells. These changes are known to have anti-apoptotic effects [58]. Sperm apoptosis is induced when the activity of both caspase 3 and caspase 9 increases, and the MMP and sperm motility decrease [59].

In the testes, Bax and Bcl-2 activities result in incomplete spermatogenesis and, ultimately, apoptosis [60]. ASTA has been reported to significantly suppress apoptosis by upregulating the expression of anti-apoptotic proteins, such as Bcl-2, and downregulating that of pro-apoptotic proteins, such as Bax, caspase 3/9, and Cyt-c, consistent with the results of the present study [61]. Therefore, we speculated that the inhibition of apoptosis by ASTA may be related to two factors. First, ASTA can improve sperm antioxidant defense, reduce ROS levels, and prevent apoptosis. Second, ASTA increases the transcript-level expression of Bcl-2 and decreases the transcript-level expression of Bax, phenomena that prevent the release of *Cyt-c* from the mitochondrial intermembrane space, thus inhibiting the caspase cascade events.

The reproductive performance of roosters is a key factor in poultry production, but fertility begins to decline after 45 weeks of age. The primary features are decreases in sperm volume, sperm concentration, vitality, and forward power, whereas lipid peroxidation and oxidative stress in sperm increase [62]. Here, we suggest that mitochondria are essential for spermatogenesis, sperm motility, and sperm apoptosis in aging roosters. First, mitochondria are one of the most important sites for the synthesis of testosterone. Dietary ASTA supplementation protects the mitochondrial structure under conditions of oxidative stress and accelerates the transfer of cholesterol from the cytoplasm to mitochondria, which is a key step in determining the rate of testosterone synthesis. ASTA promoted the expression of testosterone synthesis pathway genes and proteins and increased the level of testosterone secretion. Next, in the sperm, mitochondria are arranged at the edge of the tail microtubule, which is the organ most in need of energy because it promotes sperm swing. This suggests that mitochondria play a direct role in providing energy for sperm motility [57]. Dietary ASTA improved the activities of mitochondrial respiratory chain complexes and MMP and ensured ATP production for sperm movement. Finally, ASTA increased the antioxidant abilities of the sperm, whereas it inhibited the mitochondrial apoptotic pathway and reduced ROS levels and the expression of sperm apoptosis factors. Thus, these results indicate that dietary ASTA may improve semen quality by increasing testosterone levels and mitochondrial function in aging roosters.

## 5. Conclusions

**Figure 7:**
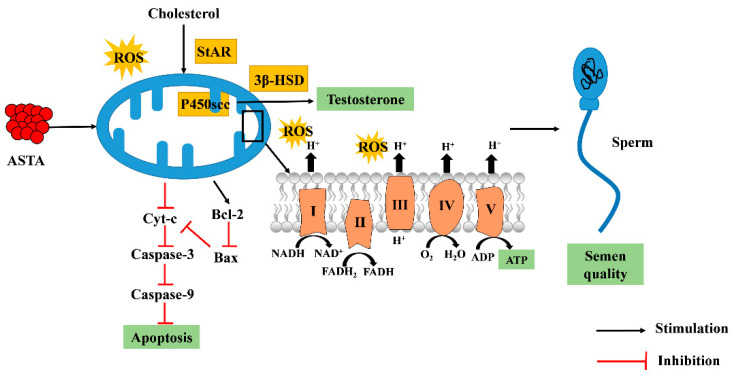
The molecular mechanism of action involves increasing sperm concentration and sperm volume in aging roosters by upregulating the testosterone synthesis pathway and improving mitochondrial function.

In summary, the current study suggests that the mechanism of action of dietary ASTA involves increasing sperm concentration and sperm volume in aging roosters (Figure 7). We speculate that dietary ASTA promotes spermatogenesis in aging roosters by decreasing the ROS levels and protecting sperm antioxidant defense systems, which may be related to increased testosterone levels and the maintenance of mitochondrial function. However, more in-depth studies on the effects of ASTA with respect to mitochondrial function are required.

## Figures and Tables

**Table 1:** Composition and nutrient content of the basal diet (air dried basis, %).

Ingredients	Content, %	Nutrition Level	
Corn	69.93	Metabolizable energy, MJ·kg^−1^	12.02
Soybean meal	18.60	Crude protein, %	14.00
Wheat bran	3.80	Methionine, %	0.23
Soybean oil	1.40	Lysine, %	0.64
Limestone	2.60	Calcium, %	1.37
Dicalcium phosphate	1.80	Total phosphorus, %	0.63
Salt	0.25	Available phosphorus, %	0.44
Choline chloride	0.20		
Premix ^1^	1.42		
Total	100.00		

^1^ The premix provided the following, per kilogram of the diet: Cu 10 mg, Fe 80 mg, Mn 100 mg, Zn 80 mg, VA 20,000 IU, VD 3000 IU, VE 30 IU, VK 2 mg, VB1 2 mg, VB2 10 mg, VB6 6 mg, VB12 0.012 mg, folic acid 1.2 mg, D-pantothenic acid 12 mg, nicotinic acid 40 mg, biotin 0.2 mg, and Se 0.3 mg.

**Table 2:** Primer sequences used for the quantitative real-time PCR.

Genes	Primer Sequence (5′-3′)	Product Size, bp	Accession Number
*Caspase-3*	Forward: TGCTCCAGGCTACTACTCCT	91	NM_20472.51
	Reverse: TTTCCTGGCGTGTTCCTTCA		
*Caspase-9*	Forward: GTCCATCCCAGTCCAACCTG	98	XM_424580.6
	Reverse: GGTACACCAGTCTGTGGTCG		
*Bax*	Forward: ACAGGGATCGTCACAGTCAT	120	XM_015290060.2
	Reverse: CACCAACTGTGTGTCGTAGG		
*Bcl-2*	Forward: TCGTCGCCTTCTTCGAGTTC	150	NM_205339.2
	Reverse: CATCCCATCCTCCGTTGTCC		
*Cyt-c*	Forward: CCTGTCCTGGTGCATGATG	77	NM_205147.1
	Reverse: TACTCTGATCCAGCTCTGCCTGAA		
*3β-HSD*	Forward: GCAAGAGGCTGGCAGAGGAATG	90	NM_205118.2
	Reverse: GGTGACGGCGTCGATGAA		
*P450scc*	Forward: GCTTTGCCTTGGAGTCTGTG	104	NM_001001756.2
	Reverse: GGTGACGGCGTCGATGAA		
*StAR*	Forward: TCAGCCGGCGGATTTAAGG	64	NM_204686.2
	Reverse: TGGTGGCTGCTACAAACACT		
*ACTB*	Forward: GCCAACAGAGAGAAGATGACAC	118	NM_205518
	Reverse: GTAACACCATCACCAGAGTCCA		

## Data Availability

The data are contain within the manuscript.

## Abbreviation

ASTA: Astaxanthin; FSH: Follicle-stimulating hormone; LH: Luteinizing hormone; T: Testosterone; GSH-Px: Glutathione peroxidase; SOD: Superoxide dismutase; CAT: Catalase; MDA: Malondialdehyde; T-AOC: Total antioxidant capacity; ROS: Reactive oxygen species; MMP: Mitochondrial membrane potential; StAR: Steroidogenic acute regulatory protein; P450scc: Cholesterol side-chain cleavage enzyme; 3β-HSD: 3β-hydroxysteroid dehydrogenase; *Cyt-c*: Cytochrome c.
